# [^99m^Tc]Technetium and Rhenium Dithiocarbazate Complexes: Chemical Synthesis and Biological Assessment

**DOI:** 10.3390/pharmaceutics17010100

**Published:** 2025-01-13

**Authors:** André Gustavo de Araujo Fernandes, Alyne Eloise Lafratta, Carolina Portela Luz, Debora Levy, Daniele de Paula Faria, Carlos Alberto Buchpiguel, Ulrich Abram, Victor Marcelo Deflon, Fabio Luiz Navarro Marques

**Affiliations:** 1 Instituto de Química de São Carlos, Universidade de São Paulo, São Carlos 13566-590, SP, Brazil; agafernandes@uesc.br; 2 Departamento de Ciências Exatas, Universidade Estadual de Santa Cruz, Ilhéus 45662-900, BA, Brazil; 3 Laboratory of Nuclear Medicine (LIM-43), Hospital das Clinicas HCFMUSP, Faculdade de Medicina, Universidade de Sao Paulo, Sao Paulo 05403-911, SP, Brazil; alyne@alumni.usp.br (A.E.L.); carol.p.luz@gmail.com (C.P.L.); danielefaria1@gmail.com (D.d.P.F.); buch@usp.br (C.A.B.); 4 Lipids, Oxidation, and Cell Biology Team, Laboratory of Immunology (LIM19), Heart Institute (InCor), Hospital das Clinicas HCFMUSP, Faculdade de Medicina, Universidade de Sao Paulo, Sao Paulo 05403-900, SP, Brazil; d.levy@fm.usp.br; 5 Institute of Chemistry and Biochemistry, Freie Universität Berlin, Fabeckstr. 34-36, D-14195 Berlin, Germany; ulrich.abram@fu-berlin.de

**Keywords:** rhenium, technetium-99m, dithiocarbazates, tumor, TM1M, B16F10

## Abstract

Background/Objectives: Dithiocarbazates (DTCs) and their metal complexes have been studied regarding their property as anticancer activities. In this work, using S-benzyl-5-hydroxy-3-methyl-5-phenyl-4,5-dihydro-1H-pirazol-1-carbodithionate (H_2_bdtc), we prepared [ReO(bdtc)(Hbdtc)] and [[^99m^Tc]TcO(bdtc)(Hbdtc)] complexes for tumor uptake and animal biodistribution studies. Methods: Re complex was prepared by a reaction of H2bdtc and (NBu_4_)[ReOCl_4_], the final product was characterized by IR, ^1^H NMR, CHN, and MS-ESI. ^99m^Tc complex was prepared by the reaction of H2bdtc and [[^99m^Tc]TcO_4_^−^ and analyzed by planar and HPLC radiochromatography, and the stability was evaluated against amino acids and plasma. Biodistribution was performed in C57B/6 mice with B16F10 and TM1M implanted tumor. Results: Re is asymmetric coordinated by two dithiocarbazate ligands, one with O,N,S chelation, and the other with N,S chelation; [[^99m^Tc]TcO(bdtc)(Hbdtc)] was prepared with a radiochemical yield of around 93%. The radioactive complex is hydrophobic (LogP = 1.03), stable for 6 h in PBS and L-histidine solution; stable for 1 h in plasma, but unstable in the presence of L-cysteine. Ex vivo biodistribution demonstrated that the compound has a fast and persistent (until 2 h) uptake by the spleen (55.46%), and tumor B16F10 and TM1M uptake is lower than 1%. In vivo SPECT/CT imaging confirmed ex vivo biodistribution, except by heterogenous TM1M accumulation but not in the B16-F10 lineage. Conclusions: H_2_bdtc proved to be an interesting chelator for rhenium or [^99m^Tc]technetium. The right spleen uptake opened the opportunity to deepen the study of the molecule in this tissue and justifies future studies to identify the reason of heterogenous uptake in TM1M tumor uptake.

## 1. Introduction

**Figure 1:**
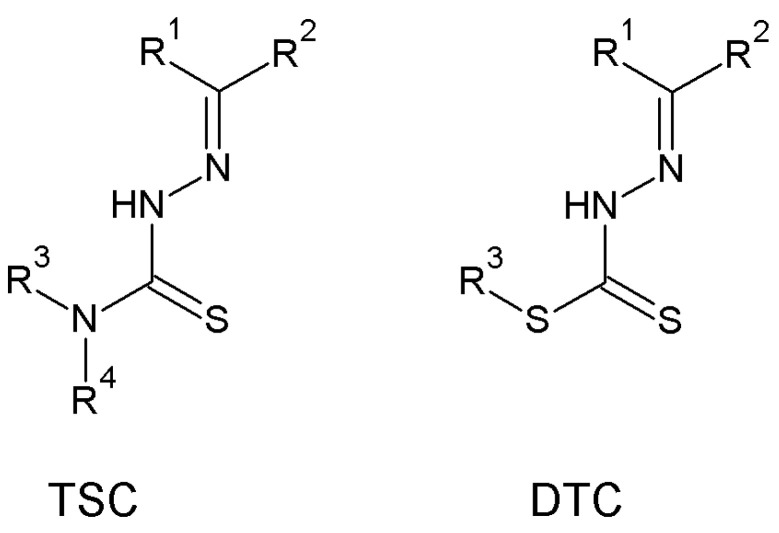
The backbone of a thiosemicarbazone (TSC) and a dithiocarbazate (DTC); R = alkyl or aryl groups.

Dithiocarbazate (DTC) and thiosemicarbazone (TSC) molecules have nitrogen and sulfur in their structure (Figure 1), which can be used to coordinate metals. DTCs or their metal complexes have been evaluated and showed activity that is trypanocidal [1], antibacterial [2,3], and antifungal [4]. The same has happened with TSCs and their complexes [5,6]. Another important application is related to cancer treatment. A large number of molecules have been evaluated as chemotherapeutics, mainly for TSCs [7,8,9] and in a minor number for DTCs [10,11,12].

A less developed application for TSCs and DTCs is as a radiotracer or radiopharmaceutical. By the introduction of radioactive metal, it is possible to image metabolic pathways using [^64^Cu]Cu-CTS (CTS = 2,3-pentanedione bis(thiosemicarbazone)) for cardiac hypoxia [13,14,15] or [^64^Cu]Cu-ATSM (ATSM = 2,3-butanedione bis(N4-methylthiosemicarbazone)) for tumor hypoxia imaging [16,17,18], the blood pool for hepatic hemangioma diagnosis using [^68^Ga]Ga-TSC-dextran [19], or tumor localization by an unknown process as reported for [^68^Ga]Ga-AATS (AATS = acetylacetate bis(thiosemicarbazones)) for fibrosarcoma [20]. Also, the radioactive complex can be used to estimate the biodistribution of non-radioactive gallium and indium complexes [21]. [^99m^Tc]technetium, one of the more exciting radiometals used in nuclear medicine, due to radioisotope characteristics such as the half-life of 6.02 h, gamma emission of 140 keV, and obtainment from a ^99^Mo/^99m^Tc generator, has been investigated for TSC and DTC radiometal complexes, such as [^99m^Tc][Tc(TSC)(CO)_3_]^+^ to be used as a precursor for bifunctional radiopharmaceuticals [22]. A nitrofuryl thiosemicarbazone complex was investigated for infection imaging [23], [^99m^Tc]technetium TSC containing stilbene and other derivatives presented affinity to amyloid-β fibers but could not cross the blood-brain barrier [24] and 4-hydroxy-pyridine derivative presented potential as an estrogen receptor linker [25]. Other [^99m^Tc]Tc-TSC complexes were prepared and evaluated for radiolabeling conditions, stability, and general biodistribution [26,27]. Less common than TSCs is the preparation of the [^99m^Tc]technetium DTC complex, with just a few reports aiming the use of S-methyldithicarbazate (NH_2_NHCS_2_CH_3_) as an intermediate to obtain a nitride [[^99m^Tc]TcN(NH_2_NHCS_2_CH_3_)] derivative [28] for further transchelation reactions [29].

Since our group has been working on the preparation of DTC metal complexes, including rhenium [1,30], we decided to investigate the radiolabeling conditions of the S-benzyl-5-hydroxy-3-methyl-5-phenyl-4,5-dihydro-1H-pyrazole-1-carbodithionate (H_2_bdtc) with [^99m^Tc]technetium and evaluate the behavior of the radiotracer in vitro and in vivo using B16-F10 and TM1M murine melanoma cells. Furthermore, we prepared the non-radioactive Re complex for structure characterization using ESI-MS, IR, ^1^H NMR, X-ray diffraction, and elemental analysis. This structurally characterized Re complex will be used to confirm the ^99m^Tc complex structure by comparing the HPLC retention time for both.

## 2. Materials and Methods

### 2.1. Materials

Chemical compounds were analytical grade (Sigma Aldrich Chemie GmbH, Schnelldorf, Germany or Merck KGaA, Darmstadt, Germany), and were used without additional purification. B16-F10 murine melanoma cells were purchased from the American Type Culture Collection (ATCC CRL-6475^TM^), and TM1M cells were selected in our institution [31]. (NBu_4_)[ReOCl_4_] is routinely prepared in our laboratory, as described in the literature [32]. The ligand H_2_bdtc was prepared as published previously [1]. [^99m^Tc]NaTcO_4_ solutions were obtained from a [^99^Mo/^99m^Tc] generator produced by the Instituto de Pesquisas Energéticas e Nucleares (IPEN, Brazil).

### 2.2. Instruments

FTIR spectra were measured as KBr pellets on an IR Prestige-21 (Shimadzu Co., Kyoto, Japan) spectrophotometer between 400 and 4000 cm^−1^. Elemental analyses were determined using the CHN EA 1108 equipment (Fisons Instruments, Rodano, Italy). ^1^H NMR spectra were acquired in the NMR Avance III instrument—400 MHz (Bruker BioSpin GMBH, Rheinstetten, Germany) at room temperature with deuterated chloroform (CDCl_3_) as the solvent. HPLC analysis was performed on a Shimadzu VP series (Shimadzu Co., Kyoto, Japan), equipped with a UV detector and connected to a radiation detector, the PerkinElmer Flow Scintillation Analyzer, Radiomatic 610RT. Planar chromatography was analyzed using a Bioscan Scanner AR2000 (Eckert & Ziegler Radiopharma, Inc., Wilmington, MA, USA) or a Perkin Elmer 1480 Automatic Gamma Counter Wallac Wizard 3” (Turku, Finland). The same gamma counter was used to measure the radioactivity in animal organs. SPECT/CT images were obtained on a Triumph SPECT/CT scanner (TriFoil Imaging, Los Angeles, CA, USA).

### 2.3. Animal Handling

The C57B/6 male mice were acquired from the Faculdade de Medicina-USP vivarium and kept in individually ventilated cages in a controlled room (20 ± 2 °C and 50–60% humidity), under a 12/12 h light/dark cycle. Food and water were provided ad libitum, except on the experiment day, when food was removed 3 h before the procedure. Animal handling procedures were carried out under anesthesia with 3% isoflurane in 100% oxygen.

### 2.4. Synthesis of [ReO(bdtc)(Hbdtc)] and Chemical Characterization

**Scheme 1:**
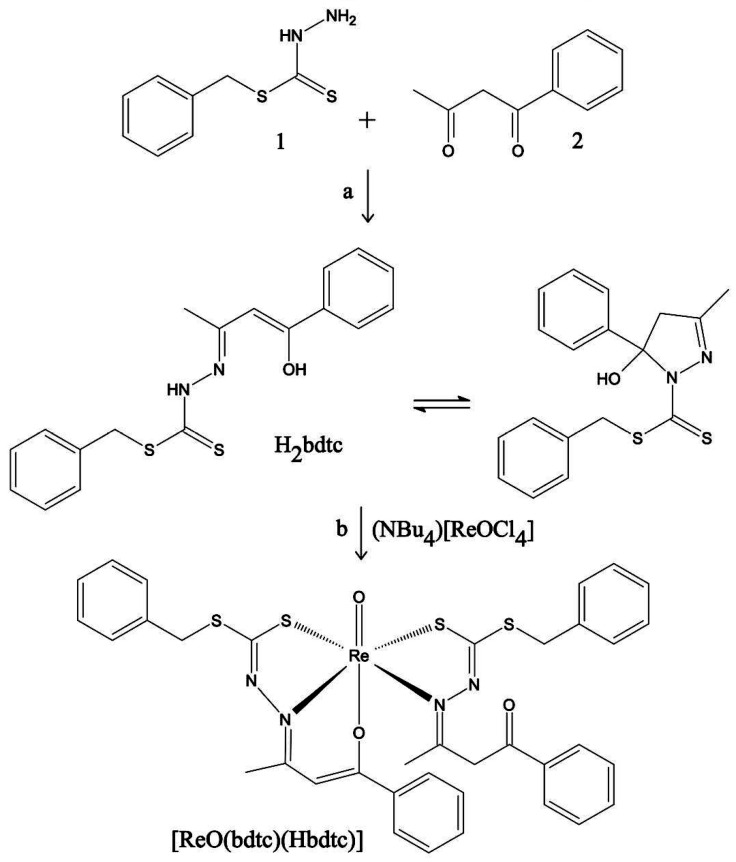
Synthesis of the ligand H_2_bdtc and [ReO(bdtc)(Hbdtc)]. Reagents and conditions. Step 1: (a) MeOH, stirring, room temperature, 8 h, and isolated; Step 2: (b) MeOH:CH_2_Cl_2_ (1:1), stirring, room temperature, 30 min.

For the preparation of the [ReO(bdtc)(Hbdtc)] complex, the ligand S-benzyl-5-hydroxy-3-methyl-5-phenyl-4,5-dihydro-1H-pyrazole-1-carbodithionate (H_2_bdtc) was prepared by the reaction of S-benzyldithiocarbazate (**1**) and 1-phenyl-1,3-butadione (**2**), as summarized in Scheme 1 [1]. The complex was obtained from mixing a solution of (NBu_4_)[ReOCl_4_] (0.058 g, 0.1 mmol) and H_2_bdtc (0.068 g, 0.2 mmol) in MeOH:CH_2_Cl_2_ (1:1, 10 mL), stirred for 30 min, resulting in a rapid color change from light yellow to dark red. Red single crystals, adequate for X-ray diffraction, were obtained by slow solvent evaporation for 2 days at room temperature. The single crystals were filtered off, washed with n-hexane, and dried under reduced pressure. Yield: 0.082 g, 93%.

The crystal structure data were collected with Mo-Ka radiation (λ = 71.073 pm) at a temperature of 200 K in an IPDS 2T diffractometer (STOE & Cie GmbH, Darmstadt, Germany). Standard procedures were applied for data reduction and absorption correction. The structures were solved by direct methods and refined with anisotropic displacement parameters using the program SHELXS-97 [33]. Idealized hydrogen atom positions were calculated with the riding model option of SHELXL-97. Crystal data and structure refinement parameters are summarized in Table 1, and selected bond distances and angles are listed in Table 2. CCDC 1549693 contains the supplementary crystallographic data for this paper.

Elemental analysis for calculated mass for C_36_H_33_N_4_O_3_ReS_4_ (884.13 g·mol^−1^): C, 48.91; H, 3.76; N, 6.34; S, 14.50. Found: C, 48.79; H, 3.45; N, 6.26; S, 14.42.

NMR spectra were obtained on a Varian Mercury plus 400 spectrometer (9.4 T field and frequency of 400 MHz for ^1^H), being internally referenced to TMS. ^1^H NMR (400 MHz, CDCl_3_-d1, δ ppm): 2.56 (CH_3b_, 3H, s), 2.98 (CH_3a_, 3H, s), 3.48 (CH_2c″_, 1H, dd, ^2^J = 13 Hz), 3.68 (CH_2a″_, 1H, dd, ^2^J = 15 Hz), 3.81 (CH_2c′_, 1H, dd, ^2^J = 13 Hz), 4.49 (CH_2b″_, 1H, dd, ^2^J = 13 Hz), 4.56 (CH_2b′_, 1H, dd, ^2^J = 13 Hz), 4.63 (CH_2a′_, 1H, dd, ^2^J = 15 Hz), 6.75 (CH, 1H, s), 7.61-6.96 (Ar, 20H, m) (Figure S2).

The mass spectra were obtained using the electrospray ionization (MS-ESI) method on an Agilent 6210 ESI-TOF spectrometer. Positive ESI-MS (*m/z*): 885.10 ([M + H]^+^), 907.08 ([M + Na]^+^), 923.06 ([M + K]^+^) (Figure S4).

FTIR spectra were measured as KBr pellets on an IR Prestige-21—Shimadzu, with elected IR data (KBr, pallets, cm^−1^): νC=N 1601, νSCS 984, νC=O 1682, νRe=O 957. Spectroscopic and analytical data for full characterization of compounds are given in the Supplementary Materials (Figure S3, Table S1).

### 2.5. Radiolabeling and Quality Control

#### 2.5.1. Preparation of [[^99m^Tc]TcO(bdtc)(Hbdtc)]

The radiocomplex was prepared by adding, in a borosilicate vial, 300 µL of nitrogenated solution of H_2_bdtc in DMSO (1 mg/mL), 300 µL of [^99m^Tc]NaTcO_4_ (370–740 MBq) solution, 20 µL of SnCl_2_ solution (2.5 mg of SnCl_2_.2H_2_O, 10 µL of HCl 37%, 10 mL nitrogenated water), and 640 µL phosphate buffer (PB) solution (0.1 M, pH 7). The vial was closed and the solution stood at room temperature for 15 min. The solution was analyzed for pH using a universal paper indicator.

#### 2.5.2. Radiochemical Purity

The radiochemical purity was assessed by planar chromatography using paper W3MM/NaCl 0.9%, W3MM/acetone systems, and by HPLC using column RP-C18 Synergy-Hydro-RP, 300 × 3.9 mm × 10 µm (Phenomenex, Torrance, CA, USA), mobile phase acetone:H_2_O:MeOH (66:22:12), isocratic model at 1 mL/min. [ReO(bdtc)(Hbdtc)] standard was co-injected with the radioactive samples in the HPLC, and the UV detector was set up to 260 nm.

#### 2.5.3. Partition Coefficient

Log P_o/w_ for the ^99m^Tc-complex was determined by adding 100 µL of the radioactive solution to a mixture of 3 mL of n-octanol and 2.9 mL of phosphate buffer (0.1 M, pH 7). The mixture was vigorously vortexed for 1 min and stood for 5 min. Aliquots of 1 mL organic and inorganic layers were removed and activity was independently determined in a dose calibrator. The partition coefficient (P) was calculated using the equation: P_o/w_ = (radioactivity in n-octanol)/(radioactivity in water), and the result was given in a logarithm value (Log P_o/w_). The results represent the mean of the three measurements.

#### 2.5.4. Complex Stability

Complex stability was evaluated for saline, L-cysteine, L-histidine, and mice plasma. In a borosilicate vial were added 100 µL of ^99m^Tc complexes and 900 µL of: (a) saline (NaCl 0.9%); (b) 1 mM L-cysteine hydrochloride solution (PBS 0.1 M, pH 7.4); (c) 1 mM L-histidine hydrochloride solution (PBS 0.1 M, pH 7.4); (d) mice plasma, isolated as described early [34]. The solutions were kept at 37 °C, sampling at times 0, 1, 3, and 6 h, and evaluated using the HPLC conditions to analyze radiochemistry purity. For a, b, and c, sampling was carried out directly from the test vials, and for d, proteins were precipitated using acetonitrile and centrifuged; the liquid phase was filtered in a 0.45 µm filter, and the sample was removed from the filtered [34]. In addition, a, b, c, and d were analyzed by planar chromatography using the systems (1) W3MM/acetone and (2) W3MM/NaCl 0.9%; in this case, proteins from plasma were not precipitated.

### 2.6. Biological Assay

#### 2.6.1. In Vitro Cell Uptake

B16-F10 and TM1M murine melanoma cell lines were maintained as monolayer cultures in RPMI-1640 medium containing 10% fetal bovine serum and penicillin (100 U/mL)-streptomycin (100 μg/mL), under 37 °C, 5% CO_2_, and humidity of 95%. For uptake studies, 1 × 10^5^ cells were plated in culture dishes (d = 16 mm) and left standing overnight for adherence. For uptake experiments, the medium was changed by a fresh one, without antibiotics, and 10 µL of a solution containing 37 KBq of the ^99m^Tc complex was added to each culture dish (n = 4/time/cell line). The incubation was maintained for 15, 60, and 120 min. At the end of each time, the medium was quickly removed, cells were washed twice with cold PBS, and these solutions were transferred to counter tubes. The cells were harvested using trypsin and transferred to counter tubes. The activity of the samples was counted in a gamma counter, and uptake yield was calculated by dividing counts in the cell tube by the total counts in the system. Cell viability was accessed by trypan blue staining.

#### 2.6.2. Ex Vivo Biodistribution

Mice were anesthetized with isoflurane at 3% in O_2_, and B16-F10 and TM1M murine melanoma cells suspended in saline were inoculated on the animal’s superior left and right flanks, respectively. The TM1M grew for 13–14 days and B17F10 for 10–12 days, or until it reached no more than 0.75 cm^3^. After tumor growth, animals were anesthetized, and 100 µL aliquot of ^99m^Tc complex (20 MBq in saline) was injected into the penile vein of male C57Bl/6 mice (8 weeks, 25–28 g). At 15, 60, and 120 min post-injection, the animals (n = 3–4), under anesthesia effect, were euthanized by heart extirpation. The blood and urine samples, tumor, muscle, bone, brain, thyroid, lungs, heart, kidneys, stomach, spleen, liver, large intestine, small intestine, and bladder were dissected and weighed; their radioactivity was measured in a gamma counter. The results expressed as a percentage of injected dose per gram of tissue (%ID·g^−1^). 

#### 2.6.3. In Vivo Biodistribution

Another group of animals (n = 2/group), presenting the same tumor, were anesthetized**,** and 28.6–44.4 MBq of ^99m^Tc complexes in 0.1 mL of saline:DMSO (5:1) was injected into the penile vein. SPECT images (64 projections × 60 s) were acquired 60 min after tracer injection using a dual-head camera with 1.0 mm five-pinhole collimators and a CZT detector system. Images were reconstructed using the OSEM method. CT images were acquired immediately before SPECT imaging to help with the best positioning of the animal in the center of the field of view (FOV) and anatomical information for fusion with SPECT images. Images were acquired using a flat-panel detector CT system with 45 kVp and 390 µA. After reconstruction, images (SPECT and CT) were fused using PMOD™ 4.0 (PMOD Technologies Ltd., Zurich, Switzerland) and analyzed.

## 3. Results

### 3.1. Synthesis of [ReO(bdtc)(Hbdtc)]

The ligand H_2_bdtc was previously prepared [1] by the reaction of S-benzyldithiocarbazate (**1**) and 1-phenyl-1,3-butadione (**2**), as summarized in Scheme 1. An equilibrium between the open-chain form (**3A**), which is appropriate for chelation, and the cycle-chain of the 5-hydroxypyrazolic form (**3B**) is possible. The [ReO(bdtc)(Hbdtc)] complex was obtained from the ligand H_2_bdtc (**3**) and the precursor (NBu_4_)[ReOCl_4_] (**4**), and the reaction was followed by a color change from light yellow to dark red.

Two dithiocarbazate ligands were coordinated to the metal center in different modes; one with dianionic tridentate O,N,S chelation (bdtc^2−^) and the other with monoanionic bidentate N,S chelation (Hbdtc^1−^). The ligand H_2_bdtc presents absorption bands at 3359 cm^–1^, ν(O–H), and no ν(N–H) band, in agreement with the cyclic structure found in the solid state, as shown in Supplementary Data (Figure S3) [1]. The ν(O–H) signal was lost in the rhenium complex, evidencing the deprotonation and thioenolization of the C=S group (Table S1) upon coordination. The thiocarbonyl nature of the free ligand in the solid state is supported by the absence of a ν(SH) 2600–2500 cm^–1^ [16]. The coordination through the deprotonated thioenolic tautomeric form of the ligand [–N=C(SH)–functionality] is commonly observed in their complexes, and it is further corroborated by the appearance of a new band in the range of 710–779 cm^–1^, characteristic of ν(C–S) [35]. The strong ν(C=N) 1630 cm^–1^ (in H_2_bdtc) is shifted to a lower wavenumber, 1601 cm^−1^, and the intensity in the corresponding complex indicates the coordination of the azomethine nitrogen to the metal ion [36,37,38]. A νC=O 1682 cm^−1^ for the complex agrees with one DTC ligand N,S coordinating in a monoanionic keto form. The bathochromic shift of νRe=O from 1002 cm^−1^ ([ReOCl_4_]^−^ precursor) to 949 cm^−1^ confirms the complex formation.

^1^H NMR spectra of the free ligand H_2_dtc were measured in CDCl_3_ solution at 400 MHz. The spectra of the ligand were predominantly observed in the cyclic tautomeric form, with an insignificant amount present in the open-ring form (Scheme 1). ^1^H NMR (ppm): 2.10 (s, 3H, CH_3_), 3.05 (d, 1H, ^2^J = 19 Hz, ring CH_2_), 3.44 (d, 1H, ^2^J = 19 Hz, ring CH_2_), 4.30 (d, 1H, ^2^J = 13 Hz, benzyl CH_2_), 4.41 (d, 1H, ^2^J = 13 Hz, benzyl CH_2_), 6.47 (s, 1H, OH), 7.24–7.41 (m, 10H, Ph). No NH resonance is observed, agreeing with the cyclic pyrazoline ligand form (Figure S1) [39]. Most of the ligand signals are shifted for the complex, except the OH and NH signals, which are absent for the complex (Figure S2), according to the infrared and X-ray data.

**Figure 2:**
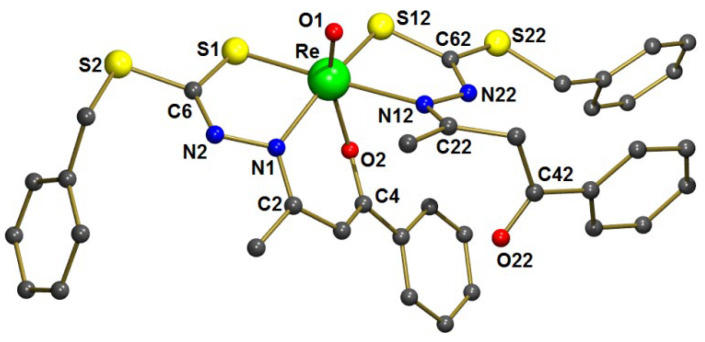
X-ray structure [ReO(bdtc)(Hbdtc)] complex. Hydrogen atoms are omitted for clarity.

The complex was analyzed by single crystal X-ray diffraction and the structure agrees with the spectroscopic results. The molecular structure of the rhenium complex with the atomic numbering scheme is shown in Figure 2 and the ortep plot is included as Supplementary Data. In addition, selected bond lengths and angles are given in Table 2. The general geometry around the metal center can be better described as a distorted octahedral coordination sphere for the rhenium atom. The metal core is coordinated with a dianionic O,N,S-tridentate facial TSC ligand, a monoanionic N,S-bidentate equatorial TSC ligand, and an oxo group at trans position to the oxygen donor atom of the tridentate TSC ligand.

### 3.2. Complex Characteristics

#### 3.2.1. Radiochemical Purity and Partition Coefficient

**Figure 3:**
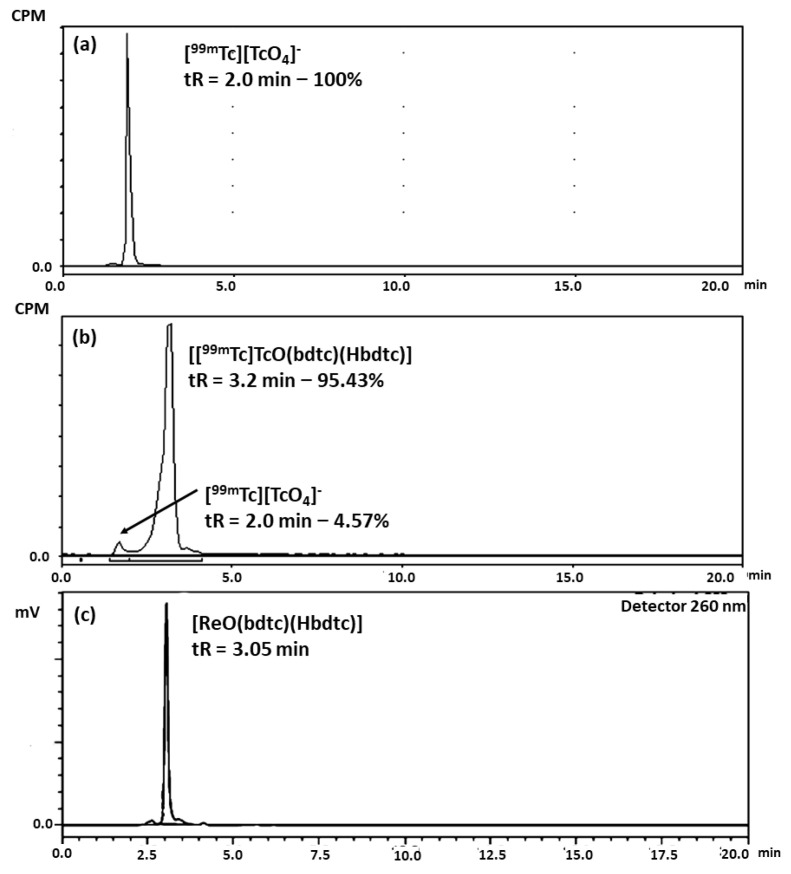
HPLC analysis for (**a**) [[^99m^Tc]TcO_4_]^−^, (**b**) [[^99m^Tc]TcO(bdtc)(Hbdtc)], and (**c**) [ReO(bdtc)(Hbdtc)].

The [[^99m^Tc]TcO(bdtc)(Hbdtc)] radiochemical purity was 93 ± 2%, as determined by reversed-phase radio-HPLC (n = 4), with a retention time (*t*R) of 3.2 min (Figure 3b), which matches the [ReO(bdtc)(Hbdtc)] standard, presenting a *t*R = 3.2–3.5 min, as observed in the UV detector (Figure 3c); unbonded [^99m^Tc][TcO_4_]^−^ reaching 5 ± 2% found at *t*R = 1.9–2.2 min, within the value found in planar chromatography (Table 3).

**Figure 4:**
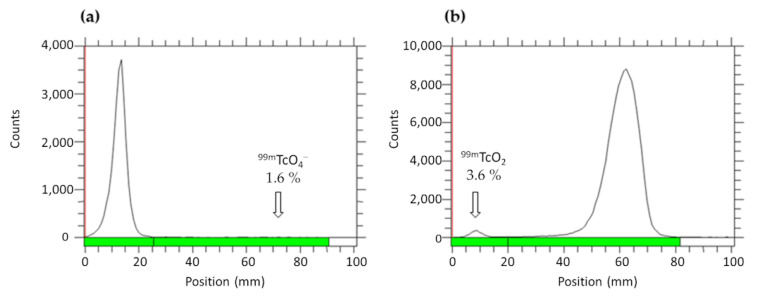
Representative planar chromatographic profile for [[^99m^Tc]TcO(bdtc)(Hbdtc)]: (**a**) W3MM/NaCl 0.9% solution and (**b**) W3MM/acetone.

Planar chromatography (PC) was performed in parallel (n = 5) and found 3 ± 1% of [^99m^Tc][TcO_4_]^−^ (Figure 4a) and 4 ± 2% for [^99m^Tc]TcO_2_ (Figure 4b).

The partition coefficient (log *p*) value was 1.03 ± 0.06 (n = 3), demonstrating the complex’s hydrophobic characteristic.

#### 3.2.2. Complex Stability

**Figure 5:**
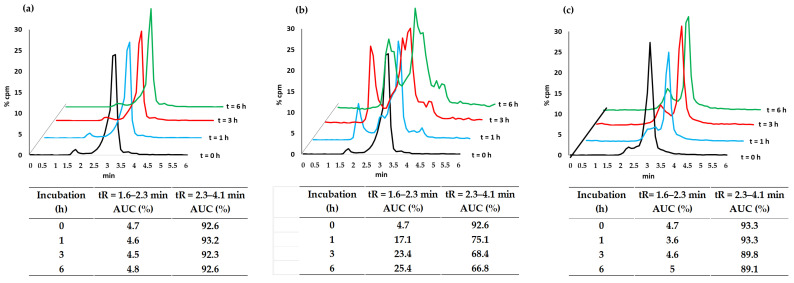
HPLC stability studies of [[^99m^Tc]TcO(bdtc)(Hbdtc)] against (**a**) L-histidine, (**b**) L-cysteine, and (**c**) plasma. *t*R = retention time. AUC = area under the curve.

[[^99m^Tc]TcO(bdtc)(Hbdtc)] was incubated in PBS (0.1 M, pH 7.4), in PBS containing 1 mM of L-cysteine (L-cys), 1 mM L-histidine (L-his), and mice plasma at 37 °C, for 1, 3, and 6 h. HPLC analysis has demonstrated the complex stability in the presence of L-his and plasma (mainly by 1 h), but in the presence of the L-cys, radiochemical purity is diminished by the decrease of the radioactivity at *t*R = 2.3–4.1 min with an increase of this one in the *t*R = 1.6–2.3 min, when compared to time 0 (Figure 5). Planar chromatographic values at Rf = 0–0.3 in the W3MM/acetone, representing [[^99m^Tc]O_2_ or [[^99m^Tc]-amino acid and plasma impurities, and at Rf = 0.7–1 in the W3MM/NaCl 0.9%, representing [[^99m^Tc]O_4_^−^ or [[^99m^Tc]-amino acid impurities obtained at time zero, were considered to compare stability. The results shown in Table 3, representing the radioactive amount in the Rf listed, demonstrated good stability for the [[^99m^Tc]TcO(bdtc)(Hbdtc)] in PBS and in the presence of L-his, since substantial changes in the values of radioactivity on the Rf analyzed during 6 h were not observed. On the other hand, the complex was unstable in the presence of L-cys by increasing the radioactivity at both selected Rf, compatible with the conversion of a hydrophobic compound to a hydrophilic one, but not [^99m^Tc][TcO_4_]^−^, since this molecule has migrated to Rf 0.7–1 in both mobile phases. In the plasma, at the system W3MM/acetone, the radioactivity concentration at Rf 0–0.3 increased during the time interval, suggesting compound protein binding. While HPLC and plana chromatography gave similar results related to the action of amino acids over the complex, the phenomena related to protein binding can not be observed in HPLC since, during the preparation of plasma sampling for this analysis, the proteins were precipitated using acetonitrile. At this point it is important to recommend the combined HPLC and planar chromatographic analysis for full characterization of radiochemical species present in the samples.

### 3.3. Biological Characteristics

#### 3.3.1. In Vitro Cellular Uptake

**Figure 6:**
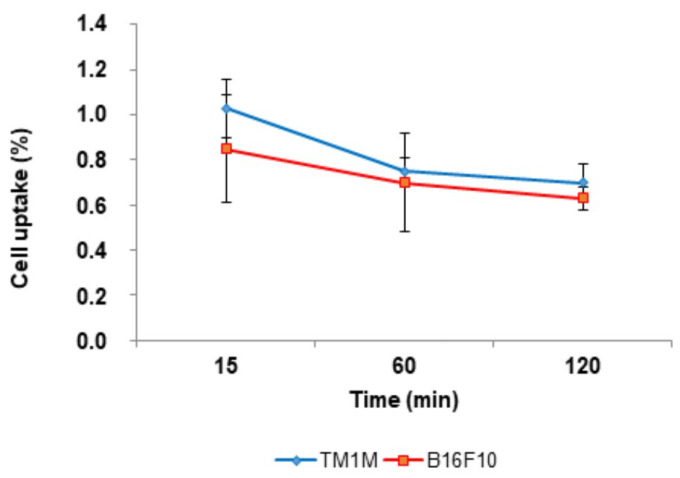
In vitro uptake of the [[^99m^Tc]TcO(bdtc)(Hbdtc)] in murine melanoma B16-F10 and TM1M culture (n = 4).

[[^99m^Tc]TcO(bdtc)(Hbdtc)] in vitro tumor uptake was evaluated for adhered B16-F10 and TM1M cells. The uptake reached 1.03 ± 0.13% and 0.85 ± 0.24% at 15 min for TM1M and B16-F10, respectively, but decreased to 0.7 ± 0.08% and 0.63 ± 0.05% after 120 min (Figure 6).

#### 3.3.2. Ex Vivo Biodistribution

The ex vivo biodistribution of the [[^99m^Tc]TcO(bdtc)(Hbdtc)] was evaluated in C57Bl/6 mice with implanted murine melanoma tumor B16-F10 and TM1M cells. The uptake value was lower than 1% ID·g^−1^ and decreased by 15, 60, and 120 min after injection, as presented in Table 4. No statistical difference (*p* < 0.05) related to all evaluated time points for each cell type was observed. Two other characteristics to be observed for this compound are the fast blood washout, with concentrations as low as 0.41 ± 0.15% ID·g^−1^ at 15 min, while at the same time, the liver uptake was 26.47 ± 4.67 % ID·g^−1^ and spleen was 63.11 ± 2.93% ID·g^−1^, still high at 120 min, as shown in Table 4. No other organ registered a noticeable uptake.

#### 3.3.3. In Vivo Biodistribution

**Figure 7:**
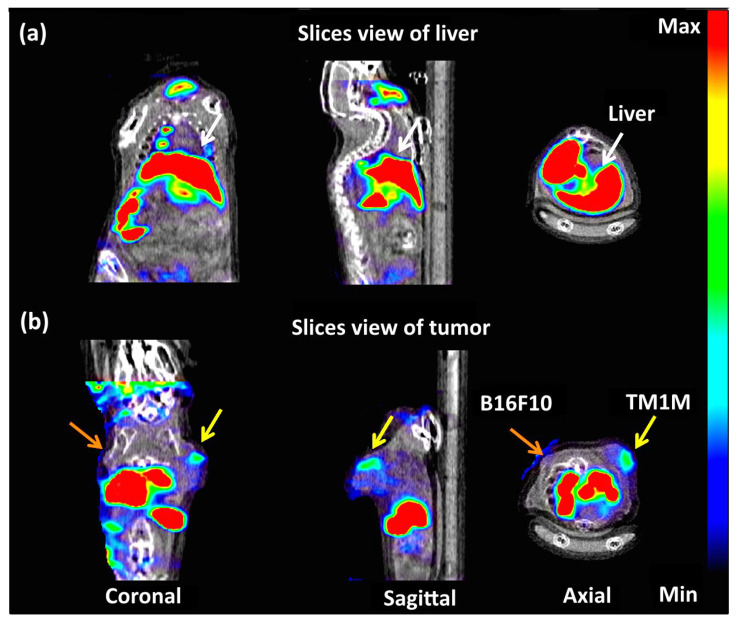
(**a**) Tomographic slices of SPECT/CT fused images of in vivo distribution of [[^99m^Tc]TcO(bdtc)(Hbdtc)] in C57Bl/6 mice in healthy mice; (**b**) tomographic slices of mice with TM1M tumors (yellow arrow) and B16F10 tumors (orange arrow). Both images were acquired 1 h after compound injection.

Biodistribution analysis was carried using in vivo imaging. Representative tomographic SPECT/CT images of the complex in the healthy animals presenting B16F10 and TM1M tumors 1 h after injection are presented (Figure 7). A high liver and spleen uptake was observed, similar to ex vivo biodistribution. However, despite the relatively low and similar in vitro uptake by both tumor cells, by image, it was possible to visualize a heterogeneous uptake of the ^99m^Tc complex in the TM1M tumor (0.75 cm^3^); in contrast, in the B16-F10 tumor (0.5 cm^3^) it was not observed. These results reinforce the importance of tomographic imaging methods in developing radiopharmaceuticals, especially over ex vivo biodistribution data. Further experiments will be planned to determine selective uptake by TM1M.

## 4. Discussion

The preparation and characterization of the [ReO(bdtc)(Hbdtc)] was necessary for structural characterization by analytical methods since it is not possible to use [^99m^Tc]technetium complexes due to the lower nanomolar concentration. The coincidence retention time for the Re and ^99m^Tc complex, as determined by HPLC, allows us to confirm the structure of the radioactive compounds.

The [[^99m^Tc]TcO(bdtc)(Hbdtc)] labeling efficiency was assessed by HPLC and planar chromatographic techniques. This second method was carried out since HPLC analysis does not allow the measurement of the impurity ^99m^Tc hydrolyzed-reduced ([^99m^Tc]TcO_2_) because it is retained in the HPLC-RP column. Technetium radiopharmaceutical purity is frequently declared as the value found during HPLC without considering the presence of [^99m^Tc]TcO_2_ species. We observed the formation was lower at around 4% for the [^99m^Tc]TcO_2_ (Figure 4b). In addition, using both chromatographic techniques allowed us to predict the radiochemical yield was around 93%.

The [^99m^Tc]technetium complex’s LogP value was 1.03 ± 0.06 (n = 3) and indicated a hydrophobic compound that can reflect high liver excretion (Figure 7, Table 4). It showed stability in PBS pH 7.4 and against 1 mM L-histidine for 6 h. However, it was unstable in 1 mM L-cysteine, which can also coordinate S,N,O-tridentate and N,S-bidentate as observed in oxorhenium(V) [40], thus competing with the DTC ligand for the metal center. In plasma, HPLC demonstrated good stability, at least by 1 h, but in planar chromatography, plasmatic protein binding was evident since the amount of radioactivity grew in the origin of the paper chromatographic system, which is characteristic behavior for radiolabeled proteins [41,42].

Tumor cell uptake was low, in vitro, and different from what occurs for several radiolabeled molecules, mainly hydrophobic compounds, which are expected to internalize in the cells by passive diffusion [43] and growth during the time. The same low uptake was observed in vivo, in part due to the fast blood washout and high liver and spleen uptake drastically decreasing the complex’s availability to be accumulated in other organs.

The ex vivo and in vivo biodistribution demonstrated a high and persistent liver and spleen uptake, with no finding of a correlation with another hydrophobic compound presented previously in the literature, such as ^99m^Tc(V)-aminooxime derivatives, with LogP = 1.35–1.43, having a maximum liver uptake of 23%; in spleen this was 1.45% ID·g^−1^ [44]; ^99m^Tc-daunorubicin, LogP = 1.7, with a liver uptake of 0.93% ID·g^−1^ and spleen 0.54% ID·g^−1^, 1 h after injection [45]; in the [[^99m^Tc]Tc(mibi)_6_]^+^, liver and spleen uptake was about 2% ID·g^−1^, 60 min post-injection, but the spleen concentration can be increased to 23.6% ID·g^−1^, when [[^99m^Tc]Tc(mibi)_6_]^+^ was encapsulated in liposomes [46]. For colloidal compounds, such as ^99m^Tc-sulfur colloidal, liver uptake is about 92.1% ID·g^−1^ between 15 min and 24 h after injections, whereas the spleen uptake was as low as 2.5 and 3.6% ID·g^−1^, during the same interval time [47]. Thus, [[^99m^Tc]TcO(bdtc)(Hbdtc)] can be a candidate for spleen imaging agents in the substitution of heat-denatured red blood cells [48,49], which involves the manipulation of the patient’s blood and reinjection, with potential risk to patient health.

Tumor uptake was relatively low as demonstrated by ex vivo and in vivo studies; however, by imaging it was possible to visualize a heterogeneous uptake of the ^99m^Tc complex in the TM1M tumor (0.75 cm^3^), whereas in the B16-F10 tumor (0.5 cm^3^) it was not observed; these findings are not uncommon for radiopharmaceuticals used in the evaluation of different tumor types, usually associated with the differential expression of some cell protein or receptor [50,51,52]. However, since ex vivo analysis did not show a difference in the tumor uptake, as observed in the image, we can consider the possibility that the tumor microenvironment components can play some role in this finding. These results reinforce the importance of tomographic imaging methods in developing new drugs or radiopharmaceuticals, especially over ex vivo biodistribution data.

## 5. Conclusions

The new [ReO(bdtc)(Hbdtc)] complex was prepared in high purity, allowing complete structural characterization that was fundamental to confirming achieved the equal compound radiolabeled with ^99m^Tc, with the match in retention time in HPLC analysis. The [[^99m^Tc]TcO(bdtc)(Hbdtc)] was also obtained in proper purity and presented limited in vitro stability that could compromise in vivo use as a diagnostic agent. However, the biodistribution data showing a fast and high liver and spleen uptake could open opportunities for developing new radiopharmaceuticals for the study of liver and spleen diseases. The lack of difference in the ex vivo TM1M and B16F10 tumor uptake, but a clear difference by the small animal SPECT imaging, can suggest that some components in microenvironments can be associated with this behavior. To deepen the biological behavior findings in this research, new projects focusing on gastroenterology and oncology need to be developed.

## Figures and Tables

**Table 1:** Crystal data and details of structural determination for the [ReO(bdtc)(Hbdtc)] complex.

Parameter Evaluation	Values
Empirical formula	C_36_H_33_N_4_O_3_ReS_4_
Formula weight	884.10
Temperature (K)	200
Wavelength (Å)	0.71073
Crystal system	Triclinic
Space group	P ī
Unit cell dimensions	
*a* (Å)	8.172(2)
*b* (Å)	15.097(4)
*c* (Å)	16.168(4)
α (°)	110.804(2)
β (°)	98.755(2)
γ (°)	95.717(2)
Volume	1.81737(8)
*Z*	2
*D* calc (g/cm^3^)	1.616
Absorption coefficient (mm^−1^)	3.614
*F*(000)	880
2*θ* range for data collection (^o^)	2.65 to 24.77
Index ranges	−9 ≤ h ≤ 9, −17 ≤ k ≤ 16, 0 ≤ l ≤ 19
Reflections collected	6120
Independent reflections	6120 [R(int) = 0.0000]
Completeness to 2*θ* = 50.00	98.3%
Refinement method	Full-matrix least-squares on F^2^
Data/restraints/parameters	6120/0/433
Goodness-of-fit on *F*^2^	1.116
*R* indices (all data)	R_1_ = 0.0573, wR_2_ = 0.0798
Final *R* indices [*I* > 2σ(*I*)]	R_1_ = 0.0387, wR_2_ = 0.0748
Largest difference peak and hole (e Å^−3^)	0.688 and −0.635

**Table 2:** Selected bond distances and bond angles for the complex [ReO(bdtc)(Hbdtc)].

Distances (pm)		Angles (°)	
Re-O(1)	168.04(4)	O(1)-Re-O(2)	156.9(2)
Re-O(2)	206.1(4)	O(1)-Re-N(1)	98.6(2)
Re-N(1)	207.1(5)	O(1)-Re-N(12)	82.9(2)
Re-N(12)	225.5(5)	O(1)-Re-S(1)	98.1(2)
Re-S(1)	230.8(2)	O(1)-Re-S(12)	102.2(2)
Re-S(12)	233.5(2)	O(2)-Re-N(1)	78.5(2)
C(4)-O(2)	130.4(7)	O(2)-Re-N(12)	75.7(2)
C(42)-O(22)	123.3(8)	O(2)-Re-S(1)	103.7(1)
C(2)-N(1)	133.0(7)	O(2)-Re-S(12)	82.9(1)
C(6)-N(2)	128.2(8)	N(1)-Re-N(12)	103.5(2)
C(6)-S(1)	175.9(7)	N(1)-Re-S(1)	79.2(1)
		N(1)-Re-S(12)	159.1(1)
		N(12)-Re-S(1)	177.0(1)
		N(12)-Re-S(12)	81.0(1)
		S(1)-Re-S(12)	96.05(6)

**Table 3:** Medium (n = 2) [[^99m^Tc]TcO(bdtc)(Hbdtc)] stability evaluated by planar chromatography.

Experimental Parameter	Rf Values	Radioactive Concentration (%) at Time			
		0	1 h	3 h	6 h
PBS 7.4	0–0.3 0.7–1	2.8 4.4	3.1 6	3 6.3	3.2 6.3
L-His	0–0.3 0.7–1	2.8 4.4	2.2 3.8	0.6 5.9	2.4 7.2
L-Cys	0–0.3 0.7–1	2.8 4.4	6.5 5.5	9 9.7	14.9 15.4
Plasma	0–0.3 0.7–1	2.8 4.4	17.7 2.4	38.6 3.0	74.5 5.2

Chromatographic systems: W3MM/acetone (Rf 0–0.3); W3MM/NaCl 0.9% (Rf 0.7–1).

**Table 4:** Biodistribution of [[^99m^Tc]TcO(bdtc)(Hbdtc)] in B16-F10 and TM1M tumor-expressing C57/Bl6 mice (n = 3), expressed as % ID·g^−1^.

Samples	15 min	60 min	120 min
Blood	0.41 ± 0.16	0.17 ± 0.04	0.17 ± 0.04
Brain	0.02 ± 0.01	0.01 ± 0	0.02 ± 0.01
Thyorid	0.51 ± 0.14	1.08 ± 0.46	0.45 ± 0.24
Heart	0.21 ± 0.06	0.2 ± 0.09	0.06 ± 0.04
Lungs	0.54 ± 0.19	0.82 ± 0.52	0.48 ± 0.24
Kidneys	0.6 ± 0.04	1.3 ± 1.04	2.15 ± 0.25
Stomach	0.7 ± 0.73	1.42 ± 0.94	1.42 ± 0.01
Spleen	63.11 ± 2.93	60.11 ± 6.74	55.46 ± 2.9
Liver	26.47 ± 4.67	29.07 ± 4.95	31.91 ± 2.73
Large intestine	0.07 ± 0	0.1 ± 0.3	0.24 ± 0.02
Small intestine	0.14 ± 0.04	0.32 ± 0.08	0.74 ± 0.1
Bone	2.58 ± 3.9	0.71 ± 0.36	3.26 ± 0.16
Muscle	0.05 ± 0.01	0.12 ± 0.15	0.04 ± 0.01
B16-F10	0.34 ± 0.07	0.31 ± 0.09	0.25 ± 0.05
TM1M	0.26 ± 0.08	0.22 ± 0.05	0.20 ± 0.06

## Data Availability

Data are contained within the article or Supplementary Material.

## Supplementary Materials

The following supporting information can be downloaded at: https://www.mdpi.com/article/10.3390/pharmaceutics17010100/s1. Figure S1: The ^1^H-NMR of the ligand H_2_bdtc; Figure S2: The ^1^H-NMR of the complex [ReO(bdtc)(Hbdtc)], CDCl_3_, 400 MHz; Figure S3. IR spectrum of the complex [ReO(bdtc)(Hbdtc)]; Figure S4: ESI-MS spectrum of the complex [ReO(bdtc)(Hbdtc)] (full and expanded area); Table S1: Important IR spectral bands (cm^–1^) and their assignments for ligand and metal complexes.
