# Supplementary material for: [99mTc]Technetium and Rhenium Dithiocarbazate Complexes: Chemical Synthesis and Biological Assessment
Source: Pharmaceutics. 2025 Jan 13;17(1):100. doi: 10.3390/pharmaceutics17010100 (PMC11768621; doi:10.3390/pharmaceutics17010100)
Supplement: Supplementary file 1 [file pharmaceutics-17-00100-s001.zip › pharmaceutics-3368149-supplementary.pdf]

SUPPORTING INFORMATION

DOI:
Title: Rhenium and [^{99m}Tc]technetium dithiocarbazate complexes: preparation and evaluation of physicochemical and biological properties
Author(s): André G. A. Fernandes, Alyne E. Lafratta, Carolina P. Luz, Debora Levy, Daniela de P. Faria, Carlos A. Buchpiguel, Ulrich Abram, Victor M. Deflon* and Fabio L. N. Marques*

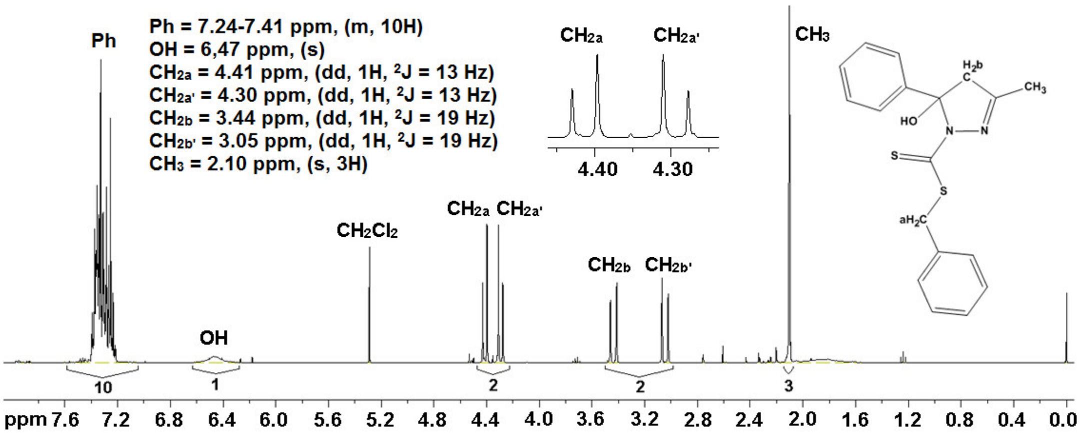

Figure S1. The ^{1}H-NMR of the ligand H_2bdtc, $CDCl_3$, TMS, 400 MHz.

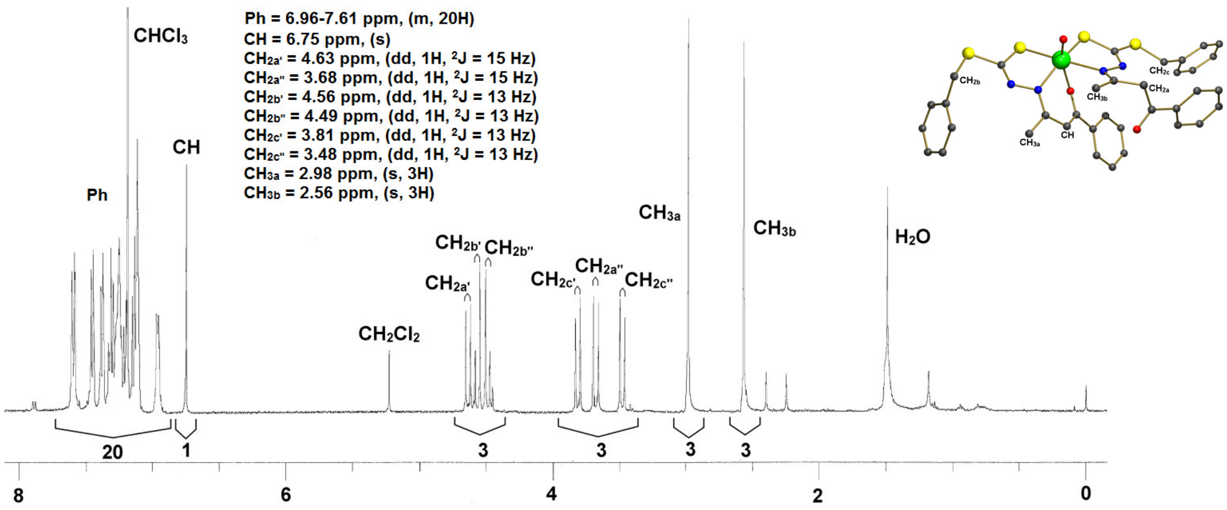

Figure S2. The ^{1}H-NMR of the complex [ReO(bdtc)(Hbdtc)], $CDCl_3$, TMS,400 MHz.

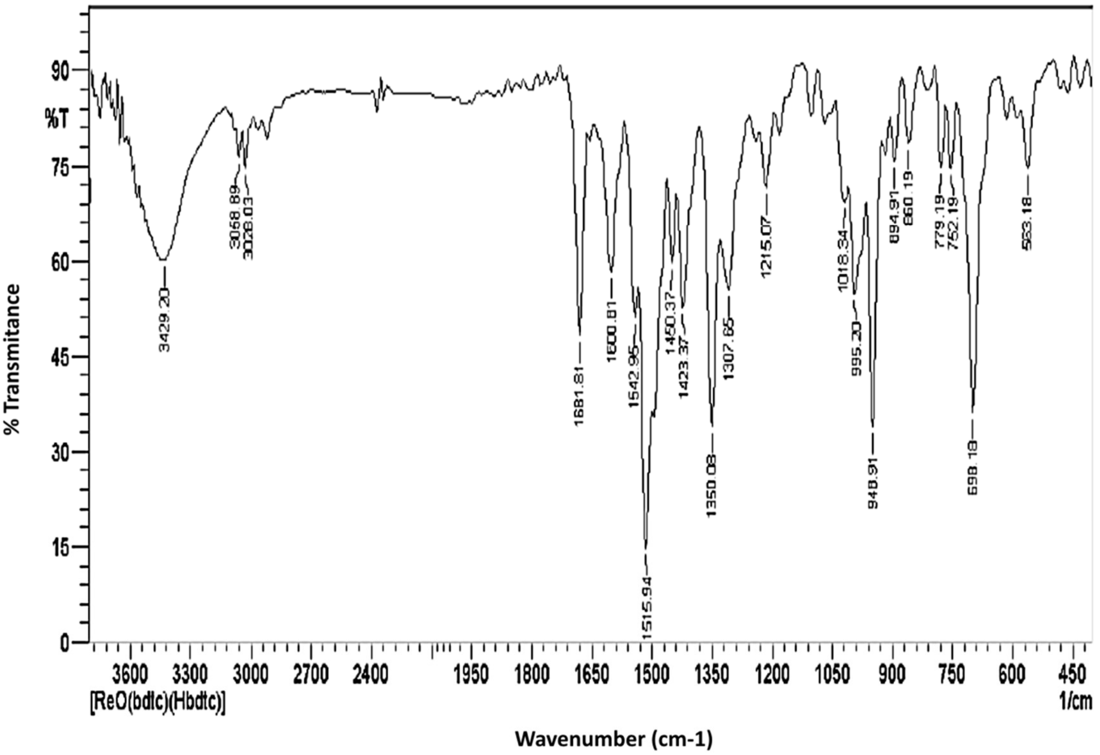

Figure S3. IR spectrum of the complex [ReO(bdtc)(Hbdtc)]

Table S1. Important IR spectral bands (cm^{-1}) and their assignments for ligand and metal complexes.

Compound	*v*(O–H)	*v*(N–H)	*v*(Re=O)	*v*(C=O)	*v*(C=N)	*v*(C=C)	*v*(C=S)	*v*(C–S)	*β(ring, Ph)*
H_2bdtc	3360	—	—	—	1629	1494, 1461	1131	854	—
(NBu_4)[$ReOCl_4$]	—	—	1002	—	—	—	—	—	—
Re complex	—	—	949	1682	1601	1547, 1516	—	750	698

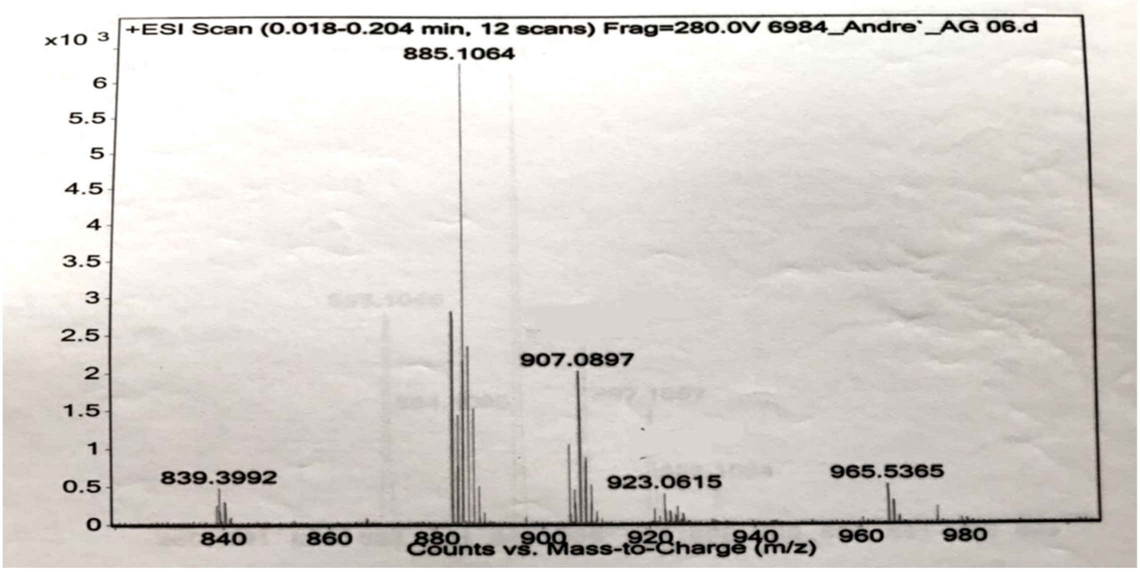

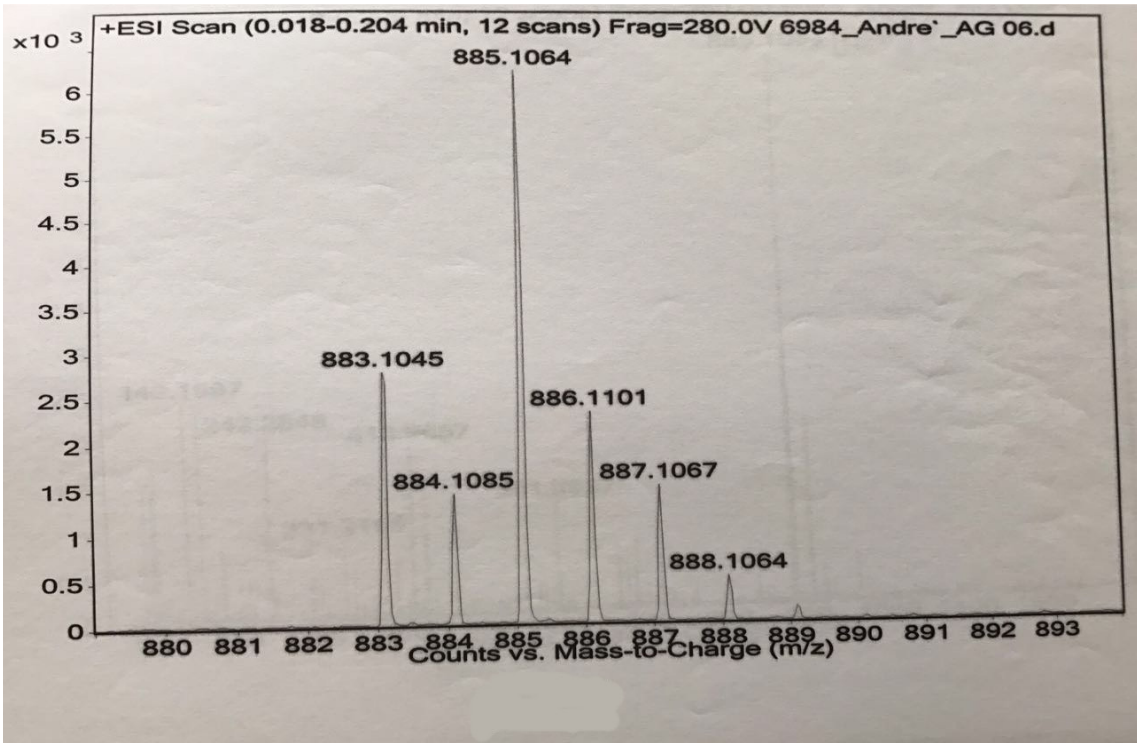

Figure S4. ESI-MS spectrum of the complex [ReO(bdtc)(Hbdtc)] (full and expanded area)
